# An Analysis of Medication Adherence in a Large Outpatient Population During the COVID-19 Pandemic Using a Novel Value-Based Pharmacy System

**DOI:** 10.1089/tmj.2023.0094

**Published:** 2024-02-07

**Authors:** Erik Hefti, Yao Xie, Kristen Engelen

**Affiliations:** ^1^ Department of Pharmaceutical Sciences, Harrisburg University of Science and Technology, Harrisburg, Pennsylvania, USA.; ^2^ RxLive, Inc., St. Petersburg, Florida, USA,; ^3^ Premier Strategy Consulting LLC, St. Louis, Missouri, USA.

**Keywords:** adherence, medication, telemedicine, telehealth, pharmacy, COVID-19, outpatients

## Abstract

**Background:**

Adherence to a medication regimen is defined as taking the medication as directed by the prescriber. Adherence is critical to achieve the desired therapeutic outcomes. Medication adherence has not been examined in large outpatient populations since the onset of the COVID-19 pandemic. A novel outpatient value-based pharmacy system (VPS) was used to collect adherence data from a large, outpatient population. The aim of this descriptive study was to analyze the reasons, medication classes, and diagnoses associated with nonadherence.

**Materials and Methods:**

Telepharmacist-documented adherence data from a large (*n* = 6,479) outpatient population that received remote consultation during the COVID-19 pandemic (August 1, 2020–November 28, 2022) were considered for this study. The adherence data were compiled within the VPS.

**Results:**

The overall rate of patients reporting at least one incident of nonadherence to their medication regimens was 21.5%. Medications used to treat hypertension, type 2 diabetes, and hyperlipidemia were least adhered to. Statins, beta-2 agonists, and corticosteroids were least adhered to. The most common reasons for nonadherence included knowledge gaps regarding therapy, forgetfulness, and side effects.

**Discussion:**

This represents the first descriptive analyses of adherence metrics in a large outpatient population during the COVID-19 pandemic. Polypharmacy, prevalence of diagnosis, and medication side effect profile may have contributed to the results observed. This study demonstrates the ability of a VPS to document key data to better inform the health care team. Elucidating adherence metrics in such populations may allow pharmacists and prescribers to identify subpopulations that require further education and management.

## Introduction

Pharmacists are health care professionals who are key members of the health care team responsible for providing information on the safe and effective use of drugs. Pharmacists provide pharmacotherapeutic guidance to patients and other members of the health care team in various clinical settings including in telehealth environments. Telehealth pharmacists (telepharmacists) routinely make recommendations that can involve the prescriber, the patient, or both. Formal recommendations that have the potential to impact patient pharmacotherapy are generally documented and supplied to the patient or prescriber in a nonstandardized format. In inpatient settings, pharmacist–provider or pharmacist–patient communications often occur through electronic health record (EHR) systems. This is not always possible in an outpatient setting. This may limit the ability of an outpatient pharmacist to effectively communicate with the patient, document interventions, and communicate with the prescriber.

Limited means of communication can reduce the participation of the pharmacist in outpatient care. Pharmacist involvement in patient care has been shown to improve various clinical outcomes and health care satisfaction metrics.^1–4^ An outpatient telepharmacy system allows for standardized communication between pharmacists, prescribers, and patients. Such a system also allows patient care information to be documented and used to improve patient outcomes via analysis of relevant metrics.^5^

One such patient metric is how often they take prescribed medication regimens as directed, or adherence. Adherence is critical for the successful delivery of pharmaceutical care and optimal clinical outcomes. High medication adherence has been associated with improved outcomes and reduced costs for multiple disease states.^6–8^ A recent meta-analysis indicated that adherence to statins has been shown to improve outcomes and reduce health care cost.^9^ Adherence to medication regimens has been associated with reduced hospitalization and health care costs in patients with chronic illnesses that include diabetes mellitus, hypercholesterolemia, hypertension, and congestive heart failure.^10^ Maintaining adherence to medication regimens can be a challenge, especially in patients with a high pill burden.^11^ Pharmacists play a key role in assessing, facilitating, and encouraging medication adherence. It has been shown that pharmacists can have a significant impact on improving medication adherence.^12^

The various methods of assessing, communicating, and documenting adherence information between the health care team in an outpatient environment can make longitudinal tracking of adherence difficult.^13,14^ Unlike an inpatient setting, patients in the outpatient setting can have multiple pharmacies and prescribers who are not under the same organizational umbrella, which can lead to gaps in communication and the inability of all members of the health care team to access documented adherence challenges. Outpatient pharmacies and prescribers often do not share a common EHR system, leading to inability to access documented pharmacist interventions for the respective patient.^15^ A value-based pharmacy system (VPS) that is adopted by the pharmacist and prescriber may fill this gap and allow for more robust adherence documentation while facilitating more effective communication of those data.

Recently, the COVID-19 pandemic has triggered an expansion of telehealth.^16^ Telepharmacy may offer a way to harmonize outpatient pharmacotherapeutic care and documentation of key clinical metrics. RxLive^®^ is an active interventional VPS developed to deliver pharmacotherapy by pharmacists remotely via modern telecommunication technology. Using this VPS, pharmacists can access and add to pertinent medical records, communicate with prescribers and patients, generate and update medication lists, and track patient adherence to prescribed medication regimes.

The VPS combines the clinical expertise of pharmacists with communications technology and data analytics to deliver value-based pharmaceutical care, with key goals of improving outcomes and lowering overall health care costs. It also allows metrics, such as adherence, to be tracked longitudinally. These data are shared between the patients and members of their health care team seamlessly. Using this VPS, participating providers can enroll their patients for a 30-min clinical consultation with a telepharmacist based on various criteria, including the number of prescribed medications.

Outpatient telepharmacists directly contact the patient via telephonic or televideo means and document all aspects of pharmacotherapeutic services rendered in the VPS. Clinically relevant information, including adherence, is then disseminated to the provider(s) and patient alike. These data can be made accessible to multiple providers in an outpatient setting without the need of sharing a common EHR. A previous study has shown the RxLive VPS was associated with reduced hospitalization in patients who had access to the service.^5^ The ability for telepharmacists to document and communicate adherence information to providers may have been a contributing factor to this, although this has not been formally explored.

The goal of this descriptive study was to characterize adherence metrics documented by outpatient telepharmacists in a large outpatient population during the COVID-19 pandemic (2020–2022). Specifically, the medications with the highest incidents of nonadherence, reasons for nonadherence, and diagnoses of patients who have high incidents of nonadherence will be explored. Elucidating these trends will allow a better understanding of baseline adherence and factors impacting nonadherence in a large outpatient population for the first time.

## Materials and Methods

### PATIENT DEMOGRAPHICS AND DATA COLLECTION

**Fig. 1.**
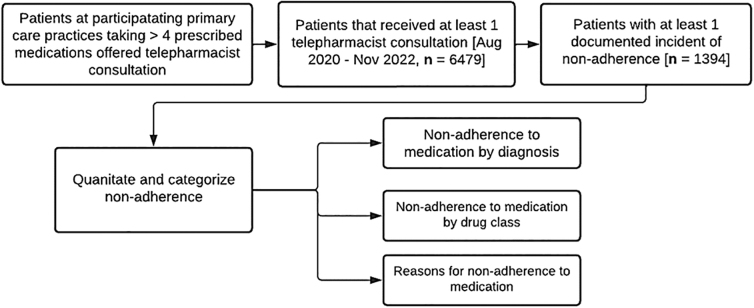
Patient selection and nonadherence quantitation workflow.

This research received a human research exemption from the IRB. Nonadherence was documented as a patient who reported not taking a prescribed medication or taking the medication in a way that deviates from the directions of the prescriber. *Figure 1* provides the sequence of patient selection and nonadherence quantitation. A total of 30 pharmacists contacted and completed a consultation with 6,479 adult patients within the collection period, from August 1, 2020 to November 28, 2022. Patients from participating primary care practices with over four medications were prompted to participate in a telepharmacy consultation with a licensed telepharmacist. Patients who completed at least one telepharmacist consult and had at least one documented incident of nonadherence were included in the adherence analysis, but some patients had multiple documented incidents of nonadherence within in a single consult or over multiple consults.

A telepharmacy consultation consists of a 30-min telephonic or video visit that includes informing the patient of the rationale for the consultation, medication reconciliation with their primary care office medication list, and screening for adherence barriers. Telepharmacists document the diagnosis for each individual medication they reconcile, as well as any patient-reported nonadherence for that medication. The telepharmacist then documents the stated reason for nonadherence specific to each nonadhered medication. Collection of data began after all participating telepharmacists underwent training on uniform intervention documentation within the VPS and were assessed for competency. The training also focused on accurate entry of diagnoses data from the patients' medical records.

Study patients were located in 22 states, with a majority located in Florida, Pennsylvania, Oregon, Arkansas, and New York. Patients were in metropolitan, suburban, and rural populations. Patient demographics are given in *Table 1*. Deidentified data on all patients receiving at least one remote pharmacist consultation and documented in the RxLive VPS were included in the analysis.

**Table 1.** Patient Demographics

	MALE	FEMALE
Total number of patients	2,701	3,778
Mean age (years)	71.1	71.7
Mean consultations received	1.3	1.3
Mean medication number	15.4	16.4
Patients with at least one incident of documented nonadherence	559	835

### DATA ANALYSIS

Prescription and over-the-counter medications were included in the current study. Vitamins and nutritional supplements were also included in this study. Adherence assessment was only performed on active, prescribed medications on patient profiles. Nonidentifiable pharmacist input was saved in tables within the RxLive internal database (RxLive, Inc., St. Petersburg, FL). The adherence data were extracted at the medication level. Each medication was also associated with a pharmacist-documented diagnosis. The data were then aggregated and analyzed in DOMO^®^ (Domo, Inc., Fork, UT) dashboards.

## Results

There were a total of 2,241 medications within the included patient population that had a documented incident of nonadherence. The incidents of nonadherence were documented by medication, with each entry representing a specific medication with a unique national drug code (NDC) number. There were 1,394 patients (21.5%) within this population who were taking at least one medication with documented nonadherence. *Table 2* provides the diagnoses associated with the highest incidents of nonadherence. The incidents of nonadherence were documented by unique NDC, with each entry associated with a patient's documented diagnosis. Different doses of the same medication would be documented individually. As patients in the population had an average of 15–16 active medications on their profile, it was possible a patient was on multiple drugs for a specific diagnosis and had multiple incidents of nonadherence documented.

**Table 2.** Diagnoses Associated with the Highest Number of Documented Incidents of Nonadherence

DIAGNOSIS	TOTAL NUMBER OF PATIENTS WITH DIAGNOSIS	INCIDENTS OF NONADHERENCE BY MEDICATION CLASS
Hypertension	4,531	299
Type 2 diabetes	1,800	196
Hyperlipidemia	3,924	173
GERD	2,192	82
COPD	656	77
Pain	1,715	52
Anxiety	1,030	42
Heart failure	358	16
Depression	241	7
Chronic kidney disease	119	6

COPD, chronic obstructive pulmonary disease; GERD, gastroesophageal reflux disease.

Patients also may, and often did, have more than one diagnosis documented. Medications used to treat hypertension, type 2 diabetes mellitus, and hyperlipidemia had the most documented incidents of nonadherence, with gastroesophageal reflux disease (GERD) and chronic obstructive pulmonary disease (COPD) also high. There were 1,291 incidents of nonadherence documented for medications with a diagnosis that fell outside of those given in *Table 2* or had an unspecified diagnosis.

*Table 3* provides the drug classes with the most documented incidents of nonadherence. The medication classified as 3-hydroxy-3-methylglutaryl coenzyme A (HMG-CoA) reductase inhibitors, corticosteroids, and beta-2 adrenergic agonists were associated with the most incidents of nonadherence. This means that different doses of the same medication within a class that are associated with nonadherence would be documented as an individual entry. There were 539 documented incidents of nonadherence documented for medications that did not have a specified drug class.

**Table 3.** Drug Classes with the Highest Number of Documented Incidents of Nonadherence

DRUG CLASS	NUMBER OF PATIENTS ON THE MEDICATIONS BY CLASS	INCIDENTS OF NONADHERENCE BY MEDICATION CLASS
HMG-CoA reductase inhibitor	3,874	149
Beta2-adrenergic agonist	1,507	105
Corticosteroid	2,098	98
Anti-epileptic agent	1,014	78
Proton pump inhibitor	2,174	78
Serotonin reuptake inhibitor	1,563	75
Beta-adrenergic blocker	2,063	66
Biguanide	1,009	65
Alpha-adrenergic blocker	1,263	56
Loop diuretic	1,065	47

HMG-CoA, 3-hydroxy-3-methylglutaryl coenzyme A.

*Table 4* provides the most commonly documented reasons for nonadherence in the patient population. The patient's lack of knowledge of their therapy, forgetfulness, and side-effects from the medication were associated with the most documented nonadherence incidents. All reasons documented were associated with a medication that had a documented nonadherence incident.

**Table 4.** Most Commonly Documented Reasons for Nonadherence

REASON FOR NONADHERENCE	PATIENTS WITH DOCUMENTED NONADHERENCE
A lack of knowledge about their therapy	542
A problem with forgetfulness	369
Manageable adverse events/side effect	309
Nonmanageable adverse event/side effect	173
Lack of motivation	146
Challenge with access to care	143
Negative beliefs about the treatment plan	113
Health literacy issue	107
Challenge with continuity of care	76
Lack of knowledge about their illness	73

## Discussion

Telehealth gained an elevated profile and increased utilization following the onset of the COVID-19 pandemic.^17,18^ The expansion of telepharmacy services presents an opportunity to better capture and track clinical metrics (such as adherence) in outpatient populations. This descriptive study demonstrates the promise of a VPS to document, track, and disseminate adherence metrics. This study considered data collected over the course of 2 years, data can be quickly collected, analyzed, and communicated to the provider for specific patients within a population, allowing more personalized care monitoring.

Although this represents a preliminary analysis, it appears that patients with hypertension, hyperlipidemia, type 2 diabetes mellitus, GERD, and COPD had the highest documented nonadherence with medications they take for those disease states. The prevalence of these diagnoses in the patient population and drug number may be a driving factor in the trends reported. The reported data trends may also be owing to the high prevalence of polypharmacy and pill burden in patients with these diagnoses.^19^ It has been previously documented that patients diagnosed with hypertension exhibited lower adherence to their regimens when they were prescribed more medications.^20^

HMG-CoA enzyme inhibitors (statins) was the drug class associated with the highest number of nonadherence incidents, which is likely owing to the high prevalence of these drugs being prescribed. Statins do have adverse effects, such as muscle aches, with variable rates of occurrence.^21^ The side effect profile of orally administered corticosteroids can be difficult for patients to tolerate, potentially leading to the trends seen with this class of drugs having many incidents of nonadherence documented.^22^ There are multiple inhaled corticosteroids reported to have low adherence in patients with asthma and COPD.^23–25^ Cost of these inhalers as well as patients not immediately feeling effects of inhaled corticosteroids may contribute to this nonadherence.^26,27^ The ability to use corticosteroid medications for a variety of respiratory and rheumatic disease states may have contributed to corticosteroids having higher incidents of nonadherence by class (*Table 2*), whereas nonadherence in COPD was lower than other diagnoses (*Table 3*).

The most reported factors impacting medication adherence in the study population included patients not understanding how to take their medication or why their medications are important, forgetting to take their medication, and experiencing side effects from their medications. Many, but not all, of the top 10 medications with nonadherence documented are taken more than once daily and have side-effect profiles that may have contributed to the trends seen. It has been previously reported that adherence decreases as administration frequency increases.^28^ Patients not understanding the importance of their medication regimens are less likely to be driven by the medications and more by the health care professionals educating them on their treatments.^29–32^ Individuals with asthma and COPD have been reported to prefer inhalers that are once-daily, potentially explaining why the nonadherence with corticosteroids and beta-2 agonist therapy was so high.^33^

The COVID-19 pandemic has resulted in higher rates of prescribing beta-2 agonists, such as albuterol, to patients without previous experience or education on the proper use of these drugs.^34^ Improved adherence to beta-2 agonists and other inhaled medications has been documented in patients with asthma during the COVID-19 pandemic.^35^ It remains unknown if beta-2 agonist-naive status impacts the adherence trends observed in the patient population.

Documenting and tracking adherence trends in large outpatient populations has traditionally been a difficult endeavor, but one that allows for exploration of key factors impacting patients' not taking medication as directed. Adherence data may provide valuable insights into key metrics within specific populations that impact the likelihood of patients complying with medication regimens. These data can help providers tailor treatment strategies to their patients for the best possible outcomes. Alternatively, adherence metrics gleaned from VPS data may provide a foundation for developing algorithmic processes to predict adherence to a prescribed medication based on patient characteristics and the medications prescribed. This predicted risk may assist with decision-making processes and allocation of health care resources (such as an appointment with a telepharmacist) to the highest risk patients. This may improve efficiency and adherence simultaneously.

Beyond a hypothetical use of adherence metrics for risk stratification algorithms, a VPS can allow for longitudinal tracking of adherence data to identify problem areas in a specific outpatient population. Because adherence can impact outcomes, the ability to reliably identify patient nonadherence may be a critical ability for provider reimbursement. If a provider can identify patients struggling with adherence, they can act earlier in the patient timeline and ameliorate the issue, hopefully before an adverse outcome occurs. Furthermore, other important data may be obtained once nonadherence is identified. Adverse drug reactions were found to be a key factor impacting adherence in this study. Obtaining this information may allow the prescriber to better tailor a drug regimen for a patient.

Utilizing a VPS allows for identifying key factors impacting adherence, as well as providing the opportunity for patient education by pharmacists. As patient knowledge gaps, beliefs, and health care literacy were identified as among the most prevalent reasons for nonadherence, telepharmacists can address these issues. Telepharmacists are well positioned to provide the needed counseling and patient education to support medication adherence. Patient education by health care professionals can impact adherence and that responsibility falls on physicians, nurses, pharmacists, and others.^36,37^ A future study will be carried out to test the hypothesis that the telepharmacist can improve adherence over time by improving patient understanding of their medications.

There are limitations in this study. Nonprescription drugs and dietary supplements were included in the active medications lists for each patient. This increased the mean number of medications per patient while often being used without prescriber direction. Adherence is difficult to assess for these agents as there is no provider-guided regimen for the patient to adhere to. As adherence is documented by individual medication, there could be specific medications at various doses responsible for the nonadherence observed. Increased resolution of which specific medications and doses are associated with nonadherence will be a component of future analyses. Documenting adherence metrics relies partially on pharmacist judgment when assigning diagnoses to medications and reasons for nonadherence from patients. This can lead to some variation in documentation. Documenting adherence based on patient reporting can also lead to some inconsistency in the data owing to the potential for patients to answer adherence questions untruthfully. When patients do not answer adherence questions honestly, it can lead to underreporting nonadherence in a population.

At present, there is only one diagnosis that can be selected for each medication the patient is taking. Some medications may be prescribed for multiple diagnoses that cannot be documented currently. The current study population, although large, is not truly heterogeneous or randomized. Most of the patients participating are older than 65 years of age and medically insured. The participating patient population was not prospectively chosen, leading to potential confounders related to age, clinical history, and willingness to participate in telepharmacy services. Patient age, education, and participation in their health care may impact outcomes and adherence, which was not controlled for in this study.^38^ Socioeconomic, race/ethnic background, and geospatial factors may impact adherence, but could not be controlled for in this study.^39–41^

The current VPS can address many of these limitations going forward. Incorporation of SureScripts^©^ (Arlington, VA) e-prescription data can be used to corroborate pharmacist documentation of adherence. SureScripts can detail the frequency that patients pick up prescriptions, adding a quantitative metric when measuring adherence beyond patient self-reporting. The integration of these data into the VPS may allow for preliminary adherence metrics to be automatically quantified for the provider before telepharmacist involvement. This will allow providers to quickly identify patients who are at high risk for nonadherence or are currently nonadherent. Wider adoption of the current VPS will allow for more diverse and heterogeneous outpatient populations to be analyzed in future studies. The VPS will have the ability to track the performance indicators and clinical utility of telepharmacist intervention in outpatient care. Utilization of a novel VPS has already been used to measure hospitalization rate of outpatient populations utilizing telepharmacy services and may have the potential to explore other outcomes in the future.^5^

## Conclusions

This study represents a preliminary examination of telepharmacist-documented medication adherence in a large, outpatient population using a novel VPS during the COVID-19 pandemic. Medications used for hypertension, hyperlipidemia, type 2 diabetes mellitus, GERD, and COPD were found to have the highest reported nonadherence, with the most cited reasons being lack of knowledge of therapy, forgetfulness, and side effects. The results suggest a VPS has the potential to enhance outpatient clinical data collection, improve the understanding of clinical trends in large outpatient populations, and the potential ability to improve overall patient care.
